# Alpha variant SARS-CoV-2 infection: How it all starts

**DOI:** 10.1016/j.ebiom.2021.103703

**Published:** 2021-11-17

**Authors:** Pere Domingo, Natividad de Benito

**Affiliations:** Department of Infectious Diseases, Hospital de la Santa Creu i Sant Pau, Institut de Recerca del Hospital de la Santa Creu i Sant Pau, Sant Quintí, 89, Barcelona 08041, Spain

Coronavirus disease 2019 (COVID-19) pandemic behaviour has been shaped by the balance between the viral evolutionary forces and the human protective effort by either non-pharmacological interventions or natural and vaccine-induced immune responses [1]. Therefore, understanding the differential attributes of the severe acute respiratory syndrome (SARS)-coronavirus (CoV)-2 variants of concern (VOC) and the host response against them may lead to a better comprehension of the complex interactions ruling the surges’ demeanour [2].

In the present issue of E-Biomedicine, Monel et al. [3] try, in a retrospective holistic study, to untangle the virologic and immunologic characteristics of SARS-CoV-2 alpha variant (B.1.1.7) compared with non-alpha variant-infected patients. They analyse 426 nasopharyngeal swabs from individuals with microbiologically proven SARS-CoV-2 infection in France: 200 from the pre-alpha dominance period and 226 from the dominant alpha surge.

The study has significant findings, including a high correlation between viral antigen detection and viable viral shedding, but a negative correlation between nasopharyngeal viral content and local immune response examined through the presence of IgA and IgG. Besides, infectious titters were associated with rapid diagnostic test (RDT) positivity, low cycle threshold (Ct) values, early time since the onset of symptoms, and absence of nasopharyngeal IgG or IgA. These findings highlight the usefulness of RDT and Ct as proxies for patient infectiousness. Since a break in the transmission chain is capital for pandemic control, the confirmation that RDT and Ct correlate with infectious viral titres is reassuring to inform checking measures.

Non-increased RNA levels on nasopharyngeal swabs of alpha variant-infected patients compared with those infected with non-alpha variants were observed in the present study. The alpha variant was also not associated with more prolonged viral shedding, and its slope of decrease was even steeper than the non-alpha variants. Furthermore, different cytokine profiles in nasopharyngeal secretions of patients infected with the alpha variant were observed. The alpha variant has increased transmissibility, leading to its global dominance after the first detection in South-Eastern England in September 2020. Mechanistic explanations for its enhanced transmissibility included higher nasopharyngeal viral load and prolonged viral shedding [4,5]. However, Monel et al. effectively demonstrate that the alpha variant does not carry higher viral RNA content or more prolonged viral shedding than non-alpha variants [3]. Therefore, other intrinsic viral features such as increased affinity for ACE2 receptor binding [5], greater fusogenicity, or the impact of sociodemographic variables must be accounted for [6,7]. Strikingly, there was no association with disease severity or patient demographics, both for either alpha or non-alpha variants. The severity of alpha variant-caused COVID-19 has conflicting data, although most authors agree that this VOC is associated with increased hospitalization rates. Most current analyses can be confounded by the impact higher transmissibility may have on hospitalization and death rates [6]. Data from the present work do not support greater pathogenicity of alpha variant despite a differential cytokine profile secretion which does not indisputably point to a hyperinflammatory profile.

Effective immune response against SARS-CoV-2 must be synchronized in space, location, amount, and quality [8]. Immune response data from Monel et al. [3] suggest that humoral response at the nasopharyngeal level is associated with markers of good prognosis. The early development of humoral response has been related to a better COVID-19 prognosis, and in mice, antibody delivery in the nasopharynx can avoid the progression of infection [9,10]. However, it is presently unknown whether IgG or IgA are locally produced or represent a spillover of a systemic response since serum immunoglobulin levels were not concurrently measured in this study.

Finally, Monel et al. [3] describe a new diagnostic technique for measuring infectious SARS-CoV-2 in nasopharyngeal swabs, the S-fuse T-cells, which is less time-consuming than the Vero E6 cells assay but equally or even more sensitive.

The present work has limitations. The paucity of clinical data exemplifies this, particularly the potential effect of comorbidities, immunocompetence, and COVID-19 treatments on viral titers, SARS-CoV-2 viability, and immune response. Moreover, having had information before the onset of symptoms would have been of great interest. Despite that, Monel's work provides information on the very early steps of SARS-CoV-2 infection, when most likely the foundations for COVID-19 severity are laid. As Plato (427 BC - 347 BC, The Republic) said, “The beginning is the most important part of the work.” For a tricky virus such as SARS-CoV-2, this is especially true.

## Contributors

Both authors coordinated and oversaw the development of the manuscript. Both authors participated in data interpretation. The manuscript was drafted by PD. Both authors provided input to the report and approved the final version of the manuscript.

## Declaration of Competing Interest

NdB does not have any relevant conflict of interest to declare. PD reports grants or contracts from Gilead, Janssen and ViiV Healthcare paid to the institution, as well as consulting fees and payments for lectures, speakers bureaus, educational events from Gilead, Janssen, ViiV Healthcare, MSD, Roche and Teravechnologies.
